# Health Outcomes in Children Associated with Prenatal and Early-Life Exposures to Air Pollution: A Narrative Review

**DOI:** 10.3390/toxics10080458

**Published:** 2022-08-08

**Authors:** Roya Gheissari, Jiawen Liao, Erika Garcia, Nathan Pavlovic, Frank D. Gilliland, Anny H. Xiang, Zhanghua Chen

**Affiliations:** 1 Department of Population and Public Health Sciences, Keck School of Medicine of the University of Southern California, Los Angeles, CA 90033, USA; 2 Sonoma Technology Inc., 1450 N. McDowell Blvd., Suite 200, Petaluma, CA 94954, USA; 3 Department of Research & Evaluation, Kaiser Permanente Southern California, Pasadena, CA 91107, USA

**Keywords:** air pollution, wildfire smoke, prenatal, early life, developmental health

## Abstract

(1) Background: The developmental origins of health and disease (DOHaD) hypothesis links adverse fetal exposures with developmental mal-adaptations and morbidity later in life. Short- and long-term exposures to air pollutants are known contributors to health outcomes; however, the potential for developmental health effects of air pollution exposures during gestation or early-childhood have yet to be reviewed and synthesized from a DOHaD lens. The objective of this study is to summarize the literature on cardiovascular and metabolic, respiratory, allergic, and neuropsychological health outcomes, from prenatal development through early childhood, associated with early-life exposures to outdoor air pollutants, including traffic-related and wildfire-generated air pollutants. (2) Methods: We conducted a search using PubMed and the references of articles previously known to the authors. We selected papers that investigated health outcomes during fetal or childhood development in association with early-life ambient or source-specific air pollution exposure. (3) Results: The current literature reports that prenatal and early-childhood exposures to ambient and traffic-related air pollutants are associated with a range of adverse outcomes in early life, including cardiovascular and metabolic, respiratory and allergic, and neurodevelopmental outcomes. Very few studies have investigated associations between wildfire-related air pollution exposure and health outcomes during prenatal, postnatal, or childhood development. (4) Conclusion: Evidence from January 2000 to January 2022 supports a role for prenatal and early-childhood air pollution exposures adversely affecting health outcomes during development. Future studies are needed to identify both detrimental air pollutants from the exposure mixture and critical exposure time periods, investigate emerging exposure sources such as wildfire, and develop feasible interventional tools.

## 1. Introduction

Air pollution is an established risk factor for morbidity and mortality that affects the general population [1,2]. The developmental origins of health and disease (DOHaD) hypothesis states that adverse fetal, infant, and childhood growth patterns are causally linked to disease development in adulthood [3,4]. Prenatal or early-childhood environmental exposures predispose the fetus or child to such mal-adaptations in growth and increase the risk of disease in adulthood, in accordance with the DOHaD hypothesis [5,6]. Two recent reviews on ambient and traffic-related air pollution have linked air pollution exposures in neonates and children with increased cardiovascular morbidity [7] and asthma development [8]. Additionally, prenatal exposure to particulate matter (PM) has been associated with higher odds of respiratory and all-cause infant mortality [9]. However, the literature on early-life air pollution exposures has not been reviewed comprehensively with respect to a broad spectrum of fetal and child health outcomes. Additionally, most reviews have focused on outdoor, ambient air pollution without specific source apportionment [7,8]. No reviews have been conducted on emerging sources of air pollution, such as traffic-related air pollution from vehicle emissions or wildfire-generated air pollution. This review aims to summarize a spectrum of health outcomes during prenatal, postnatal, and childhood development until age 10 that have been reported in recent studies, since January 2000, to be associated with early-life exposures to ambient air pollutants as well as traffic- and wildfire-generated air pollution. Given our interest in the DOHaD hypothesis, we specifically examine early-life health outcomes that are linked with lasting effects throughout development, and we categorize these into cardiovascular and metabolic, respiratory and allergic, and neurodevelopmental and psychological outcomes. Finally, we outline the biological mechanisms through which early-life exposures can impair development and health, and we summarize the links between adult-onset morbidities and adverse health outcomes during development.

## 2. Materials and Methods

We conducted a search on PubMed using the query ((air pollution) OR (air pollutant) OR (traffic) OR (wildfire)) AND ((prenatal) OR (early-life) OR (childhood)) AND ((cardiometabolic) OR (cardiovascular) OR (birth weight) OR (respiratory) OR (lung function) OR (allergic) OR (neuropsychological) OR (neurodevelopment)) to identify scientific papers related to early-life air pollution exposure and adverse health outcomes. The search included epidemiological studies published between January 2000 and January 2022, in order to include studies that examine more contemporary air pollution exposures. This search criteria resulted in 2333 journal articles with abstracts available. After discarding duplicates and reading through titles and abstracts, we selected original research studies published in English that focus on health outcomes associated with air pollution exposure during prenatal or early-childhood (0–10 years) development. The inclusion criteria for health effects were cardiovascular and metabolic, respiratory and/or allergic, and neuropsychological outcomes. Only original human studies are included in the review. We excluded studies that investigated air pollution associations beyond the stated prenatal or childhood periods, and we also excluded studies on environmental exposures not related to ambient, traffic-related, or wildfire-generated air pollution, such as heat, noise, tobacco smoke, or environmental chemicals. In addition, we examined the references of relevant papers previously known to the authors and included any relevant studies that were not found in the PubMed search. This final inclusion and exclusion criteria yielded 164 papers.

## 3. Results

Of the 164 papers included in the review, 81 examined cardiovascular or metabolic outcomes, 57 examined respiratory or allergic outcomes, and 26 examined neuropsychological outcomes associated with prenatal or early-life air pollutant exposures.

### 3.1. Cardiovascular and Metabolic Outcomes

Our search yielded 81 studies on cardiovascular and metabolic outcomes, and the results support that prenatal and postnatal air pollution exposures are both associated with an increased risk of adverse outcomes. Prenatal exposure to ambient air pollution, including particular matter with an aerodynamic diameter less than 2.5 or 10 μm (PM_2.5_ and PM_10,_ respectively), sulfur dioxide (SO_2_), nitrogen dioxide (NO_2_), or ozone (O_3_), has been consistently associated with reduced or low birth weight across various populations and geographic locations [10,11,12,13,14,15,16,17,18,19,20,21,22,23,24,25,26,27,28,29,30,31,32,33,34,35,36,37,38,39,40]. Studies that have estimated traffic-related air pollution (TRAP) or roadway proximity using geographic information system or land use regression models similarly report an association between prenatal TRAP exposure and low birth weight [12,38,41,42,43]. Prenatal exposures to ambient PM_2.5_, PM_10_, SO_2_, and O_3_ have also been associated with an elevated risk of macrosomia [44]. Although the results differ in the direction of birth weight deviation, low and high birth weight similarly reflect abnormal metabolism or nutritional transfer to the fetus, and they are both risk factors for developing cardiometabolic disorders [45,46,47,48]. Some studies have examined specific constituents of particulate matter and found that birth weight is inversely correlated with prenatal exposures to constituents, including zinc, sulfur, elemental carbon, silicon, titanium, and aluminum. [12,13,49,50] Basu et al. reported that the strongest associations were found with constituents that are common markers of traffic pollution, industrial pollution, oil combustion, and alloy production [12]. In addition to birth weight, some studies have reported that ultrasound measures of fetal growth during gestation are negatively associated with prenatal exposures to particular matter with an aerodynamic diameter less than 1 µm (PM_1_), PM_2.5_, PM_10,_ SO_2_, NO_2_, or O_3_ [22,51,52,53,54,55]. Exposures to traffic-related and ambient air pollutants, such as PM_2.5_, PM_10,_ O_3_, and NO_x_, have been consistently associated with increased odds of preterm birth [16,18,25,34,56,57,58]. However, one study did not find significant associations between NO_2_ exposure during pregnancy and preterm birth or low birth weight [59]. Early-life wildfire smoke exposure has also been associated with preterm birth and birth weight. Evidence from three studies demonstrates that pregnant women with addresses in wildfire-affected areas during gestation were at a greater risk of preterm birth or low newborn birth weight [60,61,62], while one study found a higher average birth weight in exposed male infants [63].

The results are limited on critical exposure time windows because many studies averaged air pollution exposure across an entire pregnancy or only examined exposure at one time point. Of the studies that did analyze trimester-specific associations, most found that exposures during the second [11,15,19,21,25,36,40,58,60,61] or third [11,14,16,17,31,32,33,34,36,37,39,49,58,61] trimesters had statistically significant associations with birth weight or preterm birth. A few studies report susceptibility during the first trimester to carbon dioxide (CO_2_), NO_2_, or O_3_ exposures, particularly within the first month of pregnancy [16,21,28,52]. One study that associated prenatal PM_10_ exposure with term low birth weight attributed the association to conception month and first trimester exposures [27]. One study on wildfire-related PM_2.5_ exposure found that full gestation and second trimester exposures were associated with preterm birth, while first trimester exposure was associated with decreased birth weight [60].

The literature also supports a link between prenatal air pollution exposure and abnormal weight and growth trajectory after birth. Prenatal and early postnatal exposures to ambient PM_2.5,_ PM_10,_ NO_2_, O_3_, SO_2_, and carbon monoxide (CO) have been associated with deviant growth trajectories, represented by anthropometric measures, in infancy and childhood [40,64,65,66,67,68,69,70,71]. Obesity-related parameters (higher BMI Z-score, levels of adipokines, and higher risk of obesity development) in newborns and children have been positively associated with prenatal highway proximity, TRAP exposure, or ambient PM, NO_2_, O_3_, and polycyclic aromatic hydrocarbons (PAH) exposures [67,71,72,73,74,75,76], as well as childhood exposures to TRAP and ambient PM_2.5_ and NO_2_ [77,78]. However, one study did not find an association between ambient air pollution or nearby traffic load during the first four years of life and childhood obesity, waist circumference, or cholesterol at ages four or eight [79].

Epidemiological studies also support a link between air pollution levels and the childhood risk of metabolic disorder, including diabetes and hypertension. Several studies reported that PM_2.5_ exposure during pregnancy was associated with systolic hypertension in newborns [80], and microvascular changes [81,82] and elevated blood pressure in children [83,84,85]. Prenatal TRAP, PM_2.5_, PM_10_, and NO_2_ exposures have been associated with a significant increase in cord blood insulin, adiponectin, and leptin levels, [74,75,86] with second trimester exposures having the largest effect [86]. Similarly, proximity to a major road and higher traffic-related PM_10_ and NO_2_ levels at the birth address, estimated by land use regression models, have been positively associated with childhood insulin resistance [87]. Prenatal PM_2.5_ exposure [88] and childhood TRAP exposure [89] have been positively associated with childhood development of risk factors for metabolic syndrome, such as increased hemoglobin A1c and systolic blood pressure. A study on diabetic and healthy children that were randomly selected from a pediatric database at Loma Linda University found that childhood O_3_ exposure prior to diagnosis was significantly higher in children with type 1 diabetes than in healthy controls, and pre-diagnosis PM_10_ exposure was significantly higher in children with diabetes diagnosed before age five, when compared with healthy controls [90]. In summary, prenatal and childhood exposures to ambient and traffic-related air pollution have been consistently associated with preterm birth, deviant birth weight, childhood obesity, and insulin resistance, all of which have long-term impacts on cardiometabolic health in adults (Table 1). We did not find any studies investigating early-life wildfire exposures in association with cardiometabolic outcomes in infants and children.

### 3.2. Respiratory and Allergic Outcomes

Our search resulted in 57 studies on respiratory outcomes, and the results support a link between prenatal and early-childhood air pollution exposures and respiratory morbidity. Prenatal air pollution exposure has been associated with decreased lung function during infancy and childhood [91]. Higher PM_10_ exposure during pregnancy—especially during the second [92] or third [93] trimester—was associated with worsened infant lung function, represented by increased minute ventilation, higher respiratory rate, and tidal breathing flow; in addition, preterm infants showed greater susceptibility to PM_10_-associated lung inflammation [92]. A different study reported an inverse association between CO exposure during pregnancy and infant lung function [94]. A number of studies have examined the relationship between prenatal exposure to ambient air pollution and pulmonary outcomes in childhood: prenatal exposures to ambient PM_2.5_, PM_10,_ NO_2_, NO_3,_ and benzene have been associated with worsened childhood lung function parameters, including forced expiratory volume, forced expiratory flow, airway reactance, and peak expiratory flow [95,96,97,98,99,100,101,102,103,104,105]. There is also evidence that proximity to major roads, childhood PM_2.5_ and black carbon exposures [106], and childhood NO_2_ exposure [101] is associated with worsened lung function in mid-childhood (median age 7).

The current literature presents strong evidence that prenatal air pollution exposure also increases the risk of respiratory and allergic disorders. The risk of newborn tachypnea, asphyxia, and respiratory distress has been associated with increased prenatal exposures to ambient PM, CO, NO, and O_3_ [107]. Epidemiological studies have demonstrated that prenatal exposures to ambient NO_2_, SO_2_, PM_2.5,_ PM_10_, and ultrafine particles (with aerodynamic diameter < 0.1 μm) were associated with increased respiratory tract infections in infancy [108,109,110] and asthma, wheezing, and rhinitis in childhood [109,111,112,113,114,115,116,117,118,119,120]. One study that assessed respiratory health at 6 or 18 months found no association between prenatal land use regression-modeled NO_2_ exposure and the incidence of lower respiratory tract infections or wheeze [121]. However, a different study that similarly used NO_2_ exposure estimates to quantify traffic-related air pollution reported that TRAP exposure during the third-trimester of pregnancy or first year of life was significantly associated with allergic rhinitis, and the association was strongest for male children aged 3 or 4 years old [122].

The literature also presents a consistent relationship between childhood asthma or wheeze and early-childhood exposures to ambient air pollution [102,114,119,120,123,124,125,126,127,128,129,130,131] or traffic-related air pollution, estimated by a land use regression model or road proximity [132,133,134,135]. Postnatal exposures to ambient PM_10_, NO_2_, and O_3_ have been associated with eczema and allergic symptoms in children [126,129]. Furthermore, several studies demonstrated that the risk of respiratory infection, such as pneumonia, rhinitis, or bronchitis in infants and children, was associated with increased short-term exposure to ambient PM_10_, O_3_, NO_x_, and SO_2_ [130,136,137,138], and long-term exposure to TRAP and ambient PM_2.5_, PM_10_, NO_x_, and PAH [115,129,139,140,141,142,143,144,145,146]. Still, one study did not find an association between childhood asthma incidence in kindergarten-aged children and exposure to ambient air toxics at two years, using estimates from the 2002 National Air Toxics Assessment [147]. We found only one study that examined early-life respiratory outcomes in association with wildfire-generated air pollution. This study reports an increase in respiratory visits for children aged 0–5 in association with acute PM_2.5_ exposure during a wildfire event [148]. In summary, prenatal and early-childhood exposures to TRAP and ambient air-pollution have been consistently associated with worsened lung function and asthma, wheeze, and respiratory infections in infancy or childhood (Table 2). More research is needed on early-life respiratory and allergic outcomes in association with wildfire exposures.

### 3.3. Neuropsychological Outcomes

Our literature search yielded 26 studies on neuropsychological outcomes. While early-life air pollution exposure has been less studied in children with respect to neuropsychological health, the current data suggest there is an association with adverse neurodevelopment. Prenatal and neonatal exposures to both ambient and traffic-related air pollutants, including PM, NO_2_, SO_2_, and black carbon, have been associated with impaired cognitive, motor, behavioral, and language development during infancy and early childhood [149,150,151,152,153,154,155,156,157,158,159,160]. Prenatal exposures to ambient PM_2.5_, PM_10_, and PAH have been associated with lower IQ [161,162,163] and worsened attention and memory [162] in children aged 4–7 years old. Several studies found greater odds of autism spectrum disorders (ASD) in children with higher prenatal and perinatal exposures to ambient NO, NO_2_, PM_2.5_, PM_10_, O_3_ and near-roadway air pollution, or TRAP [164,165,166,167,168,169,170,171,172]. TRAP and ambient PM_2.5_ and O_3_ exposures in the first two years of life have also been associated with an increased ASD risk [165,169,170]. Childhood exposures to near-residence traffic density, as well as the traffic-related air pollutants NO_2_, black carbon or elemental carbon, and fine and ultrafine PM, have been positively associated with cognitive and behavioral deficits, hyperactivity, and changes in white matter volume among children [156,173,174]. In summary, ambient and traffic-related air pollution exposures during pregnancy and the first two years of life have been consistently associated with ASD and worsened neuropsychological parameters, including motor and cognitive development (Table 3). Fewer studies have examined the neuropsychological outcomes associated with childhood air-pollution exposures, and no studies have examined the neuropsychological outcomes in association with early-life wildfire exposure.

## 4. Discussion

Our review indicates that early-life exposures to ambient and traffic-related air pollutants are associated with an increased risk of unfavorable developmental outcomes; prenatal and early-childhood exposures to outdoor air pollutants, including PM_2.5_, PM_10_, NO_x_, and O_3_, have been consistently associated with adverse cardiovascular and metabolic, respiratory and allergic, and neurodevelopmental outcomes in early life.

Experimental studies elucidate that early-life air pollutant exposures can increase morbidity through inflammation, oxidative stress, and transcription regulation. During gestation, air pollutants such as PM_2.5_ and PM_10_, as well as maternal inflammatory cytokines induced by air pollutants, can cross the placenta and induce fetal inflammation and oxidative stress that can last through childhood [175,176,177,178,179]. Early postnatal exposure to air pollutants such as PM_2.5_ can also induce developmental dysfunction through the generation of reactive oxygen species (ROS) and inflammatory stress [180,181], as well as epigenetic alterations [182,183,184,185]. Recent studies have found abnormal immune profiles in newborns and children exposed to air pollution in utero [186,187,188] or during childhood [186,189].

There is substantial evidence in support of the DOHaD hypothesis linking adverse fetal and infant development to metabolic, cardiovascular, and respiratory morbidity in children and adults [45,190,191]. Both low and high birth weight are known risk factors for adult diseases including obesity, hypertension, heart disease, and adult-onset diabetes [47,48,192]. Abnormal fetal growth and rapid childhood growth trajectories have also been associated with adult cardiovascular, metabolic, and respiratory morbidity [193,194,195]. The literature also supports a link between impaired respiratory health during early development and adult respiratory morbidity such as chronic obstructive pulmonary disease (COPD) [195,196,197,198]. Similarly, there is evidence supporting the developmental origins of neurological deficits and mental health illnesses in adulthood [199,200,201,202,203]. Taken together, the literature in support of DOHaD and the literature we review on early-life air pollution exposures suggest that air pollution exposure during critical developmental periods is a risk factor for adverse health outcomes later in life.

The most widely studied outcomes in our search include birth weight in association with prenatal exposure, and asthma or wheeze in association with prenatal and postnatal exposures. However, much of the data combines respiratory and allergic outcomes, such as asthma, wheeze, or rhinitis. Future research should differentiate between allergic and non-allergic respiratory symptoms, and include non-respiratory immune disorders such as dermatitis. There is also a need for more air pollution research on a wider range of neuropsychological outcomes. Most studies examined ASD diagnosis or cognitive and motor deficits; therefore, studies on psychological outcomes such as anxiety-like and depressive behaviors in young children are warranted. Additionally, while some studies have included prenatal time windows of susceptibility, critical exposure periods from gestation to childhood have not yet been well-established. This may be due in part to difficulty collecting longitudinal data on air pollution exposure, as well as difficulty routinely collecting data on potential confounders that vary during postnatal and childhood development. Still, further research should be conducted to determine the sensitive exposure time windows during early-life development, in order to better inform the DOHaD approach and target the early prevention of adverse health outcomes.

The air pollutants we found to be most consistently studied are particulate matter (PM_2.5_ and PM_10_), NO_2_, and O_3_. Other air pollutants such as black carbon or elemental carbon, CO, SO_2_, and source-specific air pollutants have been less frequently examined and warrant focused studies in the future. Additionally, identifying detrimental air pollutants from the exposure mixture and identifying the detrimental chemical components within the pollutants, in the cases where there are specific drivers of toxicity, will help inform intervention targets.

Most early-life exposures studies have focused on ambient and traffic-related air pollution, whereas few studies have been conducted on wildfire exposures. The majority of research on early-life wildfire exposure examined the associations of prenatal wildfire exposure with preterm birth and birth weight [60,61,62,63], and only one study assessed the respiratory effects of wildfire smoke exposure during early childhood [148]. While this deficit may be due to the challenges inherent in exposure assessment for wildfire-related air pollution, many studies have been conducted on wildfire smoke exposure in adults; they are, therefore, feasible to investigate in a younger population. Wildfire smoke contributes to a significant increase in exposures to air pollutants; in addition, concerns over wildfires and wildfire-associated morbidity have increased in recent years, in part due to climate change impacts. Three review articles have established a consistent link between air pollution levels during wildfire events and acute respiratory morbidity in adults [204,205,206]. Proximity to wildfire has been strongly associated with increased respiratory symptoms, medication use, or hospital visits in adults [207,208,209,210,211]. Elevated levels of ambient PM_2.5_, PM_10_, or O_3_ during a wildfire have been associated with increased adult respiratory morbidity for outcomes such as asthma, wheezing, COPD, and respiratory infection [212,213,214,215,216,217,218,219,220,221,222,223,224,225,226,227,228,229]. Epidemiological studies have reported that increased smoke or ambient PM_2.5_ exposure during wildfire are linked to increased cardiovascular events, such as hypertension, angina, and cardiac arrest, particularly among elderly [213,217,220,230,231]. The literature on early-life air pollution exposure, which we review above, coupled with the data on wildfire smoke exposure throughout the lifetime, together motivate a need for future studies to explore the adverse health effects associated with prenatal and early-childhood exposures to wildfire-generated air pollutants. Although wildfire smoke contains various particulate and gaseous pollutants found in traffic-related or ambient air pollution, it is composed of a different chemical mixture [232,233]; moreover, recent studies suggest that PM from wildfire smoke may be more toxic than equal amounts of PM from other sources [214,234,235]. As such, future studies are needed to identify the detrimental compounds specific to wildfire smoke and investigate their mechanisms of pathophysiology.

## 5. Conclusions

In conclusion, the current literature supports an association between prenatal and early-childhood exposures to air pollutants (especially particulate matters, NO_2_, and O_3_), and adverse cardiovascular and metabolic, respiratory and allergic, and neurodevelopmental health outcomes during gestational and childhood development. These air pollution-associated health outcomes during early life can predispose individuals to morbidity in adulthood, in accordance with the DOHaD hypothesis. Critical exposure time windows during development should be further clarified, possibly through natural experimental or interventional studies. There is also a gap in the research identifying specific PM components that are detrimental and exploring their individual effects on development. In addition, while there is substantial data on early-life ambient and traffic-related air pollution exposures, further research is needed to examine the developmental health effects associated with wildfire-generated pollution exposures, given the recent increase in wildfire events and the concern over climate change. Identifying causal determinants of developmental health outcomes in early life, such as air pollution exposure, will help the early prevention of chronic diseases in the general population.

## Figures and Tables

**Table 1:** Cardiovascular and Metabolic Outcomes Associated with Early-Life Air Pollution Exposures.

Health Outcome	Population Age	AP Exposure	Exposure Time Window	Association	References
Intrauterine growth restriction	Newborn	PM_2.5_, PM_10_, SO_2_, NO_2_, O_3_	Prenatal	↓ Birth weight	[10,11,12,13,14,15,16,17,18,19,20,21,22,23,24,25,26,27,28,29,30,31,32,33,34,35,36,37,38,39,40]
	Newborn	PM constituents	Prenatal	↓ Birth weight	[12,13,49,50]
	Newborn	TRAP	Prenatal	↓ Birth weight	[12,38,41]
	Newborn	Wildfire smoke	Prenatal	↓ Birth weight	[60,61,62]
	Newborn	NO_2_	Prenatal	No association with birth weight	[59]
	Newborn	PM_1_, PM_2.5_, PM_10_, SO_2_, NO_2_, O_3_	Prenatal	↓ Fetal ultrasound measurements	[22,51,52,53,54,55]
Macrosomia	Newborn	PM_2.5_, PM_10_, SO_2_, O_3_	Prenatal	↑ Birth weight	[44,66]
	Newborn	Wildfire proximity	Prenatal	↑ Birth weight	[63]
Preterm birth	Newborn	PM_2.5_, PM_10_, O_3,_ NO_x_	Prenatal	↑ Odds of preterm birth	[16,18,25,34,56,57]
	Newborn	TRAP	Prenatal	↑ Odds of preterm birth	[58]
	Newborn	Wildfire smoke PM_2.5_	Prenatal	↑ Odds of preterm birth	[60]
	Newborn	NO_2_	Prenatal	No association with preterm birth	[59]
Deviant growth trajectory	0–6 years	PM_2.5_, PM_10_, NO_2_, O_3_, SO_2_, CO_2_, CO	Prenatal	↑ or ↓ Anthropomorphic measures	[40,64,65,66,67,68,69,70,71]
	0–12 months	CO, PM_2.5_	Postnatal	↓ Anthropomorphic measures	[70]
Obesity and metabolic disorder	0–12 months	PM, NO_2_, O_3_	Prenatal	↑ BMI, ↑ fat mass, fat mass rate of change, ↑ weight for length	[67,71]
	4–14 years	PM_2.5_, O_3_, PAH	Prenatal	↑ BMI, ↑ fat mass	[72,76]
	0–9 years	TRAP, traffic proximity	Prenatal	↑ Fat mass, ↑ overweight risk	[67,73,76]
	6–11 years	PM_2.5_, NO_2_, elemental carbon	Childhood	↑ BMI, ↑ overweight or obese risk	[77,78]
	6–10 years	TRAP, traffic proximity	Childhood	↑ Overweight or obese risk, ↑ hemoglobin A1c, ↑ blood pressure	[78,89]
	4, 8 years	Traffic proximity, ambient AP	Childhood (0–4)	No association with obesity, waist circumference, or cholesterol	[79]
	Newborn	PM_2.5_	Prenatal	↑ Systolic hypertension	[80]
	4–6 years	PM_2.5_	Prenatal	↑ Microvascular changes	[81,82]
	3–9 years	PM_2.5_	Prenatal	↑ Blood pressure	[83,84,85]
	Newborn	PM_2.5_, PM_10_, NO_2_	Prenatal	↑ Insulin, ↑ adiponectin, ↑ leptin	[74,86]
	Newborn	TRAP	Prenatal	↑ Adiponectin, ↑ leptin	[75]
	10 years	TRAP and traffic proximity	Prenatal	↑ Insulin resistance	[87]
	4–6 years	PM_2.5_	Prenatal	↑ Hemoglobin A1c	[88]
	0–5 years	O_3_, PM_10_	Childhood	↑ Diabetes	[90]

↑: increasing, ↓: decreasing.

**Table 2:** Respiratory and Allergic Outcomes Associated with Early-Life Air Pollution Exposures.

Health Outcome	Population Age	AP Exposure	Exposure Time Window	Association	References
Lung function	5–9 years	PAH	Prenatal	↓ FEV1	[95]
	5–7 years	Near-roadway air pollution (NRAP), TRAP	Postnatal	↓ FVC, ↓ FEV1	[96,101,106]
	2–10 years	NO_2_, PM_10_, PM_2.5_, NO_3_	Prenatal	↓ FVC, ↓ FEV1, ↑ respiratory resistance, ↓ respiratory reactance	[97,98,99,100,103,104,105]
	2–10 years	Household air pollution	Postnatal	↑ Airway reactance, ↓ FEV1	[99,102]
	30 days–1 year	PM_10_, CO, NO_2_, O_3_	Prenatal	↑ Fractional exhaled NO, ↓ peak tidal expiratory flow, ↑ respiratory rate, ↑ minute ventilation	[92,93,94]
Respiratory tract infections	12–18 months	NO_2_ and PM_2.5_	Prenatal	↑ Lower respiratory tract infections, ↑ LRTI hospitalizations	[108,110]
	0–5 years	PM_2.5_, PM_10_, NO_x_, O_3_, SO_2_	Postnatal	↑ Respiratory infections, ↑ bronchitis, ↑ LRTI hospitalizations	[130,136,137,138,139,140,141,142,143,146]
Asthma and allergic disorders	Newborn	PM_2.5_	Preconception	↑ Transient tachypnea, ↑ asphyxia, ↑ respiratory distress syndrome	[107]
	0–10 years	SO_2_, NO_2_, PM_10_, PM_2.5_, black carbon, CO, ultrafine particles, regional NO_2_	Prenatal	↑ Wheeze, ↑ asthma	[109,111,112,113,114,115,116,117,118,119,120,121]
	3–6 years	PM_2.5_, PM_10_, NO_x_, PAH, SO_2_	Postnatal	↑ Allergic symptoms, ↑ allergic rhinitis, ↑ eczema, ↑ asthma	[102,114,115,119,120,123,124,125,126,127,128,129,130,131,144]
	0–10 years	TRAP	Postnatal	↑ Asthma, ↑ asthma hospitalizations	[122,132,133,134,135,145]
	0–5 years	Ambient air toxics	Postnatal	No association with asthma	[147]
	0–5 years	Wildfire-generated air pollution	Postnatal	↑ Respiratory hospital visits	[148]

↑: increasing, ↓: decreasing.

**Table 3:** Neuropsychological Outcomes Associated with Early-Life Air Pollution Exposures.

Health Outcome	Population Age	AP Exposure	Exposure Time Window	Association	References
Impaired cognitive development	0–2 years	PM_2.5_, PM_10_	Prenatal	↓ Cognition Score, ↓ Mental Developmental Index, ↓ Problem Solving Score	[151,153,155,160]
	4–7 years	NO_2_, PM_2.5_, PM_10_, PAH	Prenatal	↓ Global Cognition Score, ↓ IQ Score, ↓ Verbal IQ Index	[151,161,162,163]
Impaired motor development					
	0–9 years	PM_1_, PM_2.5_, PM_10_, NO_2_, NO_x_, SO_2_, iron (PM_2.5_ constituent)	Prenatal	↓ Fine Motor Score, ↓ Global Motor Score, ↓ Psychomotor Developmental Index	[149,150,154,155,157,158,160]
Impaired behavioral development	0–2 years	PM_1_, PM_2.5_, PM_10_, NO_2_, SO_2_	Prenatal	↓ Personal-Social Score, ↓ Adaptability Score, ↓ Social-Response Score	[155,158]
	2–6 years	PM_2.5_, PM_10_, NO_2_, SO_2_	Prenatal	↓ Inhibition, ↓ impulsivity, ↓ emotion expression, ↑ reported behavioral problems	[150,152]
	0–3 years	NO_2_, SO_2_	Prenatal	↓ Adaptive-Behavior Score, ↓ Social-Behavior Score	[157]
	6–10 years	TRAP, black carbon	Childhood	↑ Behavioral problems	[156]
Impaired language development	0–2 years	PM_2.5_	Prenatal	↓ Communication Score	[155]
	2–6 years	PM_2.5_, PM_10_, NO_2_	Prenatal	↓ Sentence completion, ↓ Verbal Score	[150,151]
	0–2 years	PM_1_, PM_2.5_, PM_10_, NO_2_, SO_2_	Prenatal	↓ Language Score	[157,158,159]
Attention and memory deficit	2–7 years	PM_2.5_, NO_2_	Prenatal	↓ Memory Score, ↑ omission errors, ↓ Hit Reaction Time, ↓ general memory, ↓ visual memory	[151,162]
	7–11 years	TRAP	Childhood	↓ Working memory, ↓ memory span length, ↑ inattentiveness	[173,174]
Autism spectrum disorders	2–5 years	TRAP, NRAP, freeway proximity	Prenatal	↑ ASD risk	[164,165,172]
	2–10 years	PM_2.5_, PM_10_, NO_2_, NO, O_3_, CO	Prenatal	↑ ASD risk	[165,166,167,168,169,170,171]
	2–5 years	TRAP	Childhood	↑ ASD risk	[165]
	2–10 years	PM_2.5_, PM_10_, NO_2_	Childhood (0–2 years)	↑ ASD risk	[165,169,170]

↑: increasing, ↓: decreasing.

## Data Availability

Not applicable.
